# Supplementary material for: Skin Manifestation of SARS-CoV-2: The Italian Experience
Source: J Clin Med. 2021 Apr 8;10(8):1566. doi: 10.3390/jcm10081566 (PMC8068198; doi:10.3390/jcm10081566)
Supplement: Supplementary file 1 [file jcm-10-01566-s001.pdf]

Table S1. Clinical reports of cutaneous manifestations of SarS-CoV -2 in Italy.

Author, Publication Year	Patients with Skin Signs (n)	Location	Signs
Recalcati, May 2020 [12]	18	Trunk	Erythematous rash (14 patients)<br>Widespread urticaria (3 patients)<br>Chickenpox-like vesicles (1 patient)
Mazzotta et Al., 2020 [13]	ND	Feets	Erythematous, cyanotic, purpuric, bullous and necrotic lesions
Gianotti et Al., Sachdeva M et Al. [16-17]	3	Arms, trunk and lower limbs (1 patient)<br>Trunk and arms (1 patient)<br><br>Widespread (1 patient)	Erythematous macules<br><br>widespread pruritic eruption of erythematous macules and papules
Gianotti et Al. May, 2020 [18-19]	5	1 patient:Trunk and limbs<br><br>3 patients: Trunk<br><br>1 patient: not reported	Exanthema<br><br>Papular erythematous exanthema, and diffuse maculopapular eruption clinically suggestive for Grover disease (2 patients)<br><br>Purpuric maculo-papulo-vesicular rash (1 patient)<br><br>Papular erythematous exanthema
Tosti et Al., May 2020 [20]	4	2 patients: heels<br>2 patients: toes	erythematous plaques<br><br>erythematous plaques
Colonna et Al. [21]	4	Feet	erythematous macules

**Genovese et Al. May 2020 [22]**	**1**	**Generalized**	**Generalized, pruritic morbilliform rash (petechial and erythematous maculopapular lesions)**
M. El Hachem May 2020 [23]	19	Feet	Childblain lesions
Marzano et Al, June 2020 [24]	22	Trunk	Diffuse papulovesicular lesions (predominance of papules or vescicles)
Diotallevi et Al. June 2020 [25]	3	Trunk (1 patient)<br>Trunk and abdomen (1 patient)<br>Feet (1 patient)	Erythematous orticarioid macules<br><br>Childblain lesions
**Tammaro et Al., July 2020 [26]**	**2**	**Trunk**	**Isolated herpetiform lesion**
Tammaro et Al., July 2020 [27]	1	Limbs<br>Acral sites	Erythematous lesions<br>Necrotic lesions
Piccolo et Al., July 2020 [28]	63	Acral lesions	Chilblain-like lesions
Gianotti et Al., August 2020 [29]	3 groups of patients	Various localization	Erythematous plaques, rash, acral eruption of papules and macules
Recalcati et Al., August 2020 [30]	14	Feet (8 patients)<br>Hands (4 patients)<br>Both (2 patients)	acral eruption of erythemato-violaceous papules and macules
De Giorgi et Al., August 2020 [31]	17	Widespread	Urticarial rash
Castelnovo et Al., August 2020 [32]	1	Acral	Vasculitis lesions
Maniaci et Al., August 2020 [35]	1	Lower limbs	Erythematous skin lesions
Freeman et Al., August 2020 [36]	1	Lower limbs	Erythema pernio-like lesions
A G Locatelli et Al. August-September 2020 [33]	1	Feet	Childblain lesions

Colonna et Al. September 2020 [34]	30	Chilblain-like acral lesions	Chilblain-like acral lesions
Brazzelli et Al., September-October 2020 [37]	8	Widespread	Urticarioid eruptions
Guarneri et Al., September 2020 [38]	13	Widespread<br>Widespread<br>Widespread<br>Feet	Urticarial eruptions (2 patients)<br>Panniculitis (3 patients)<br>Erythematous rash (2 patients)<br>Childblain lesion (1 patient)
Gaspari et Al., October 2020 [39]	18	Widespread<br>Acral<br>Trunk<br>Trunk	Exanthematic rashes (9 patients)<br>Acral vasculitic eruptions (6 patients)<br>Polymorpho-like urticaria (2 patients)<br>Varicelliform eruption (1 patient)
Di Nunno et Al., October 2020 [40]	34	Widespread	Skin dryness (15 patients)<br>Irritant contact dermatitis (5 patients)<br>Seborrheic dermatitis (4 patients)<br>Morbilliform rashes (4 patients)<br>Petechial rashes (3 patients)<br>Widespread hives (3 patients)
Caputo et Al., October 2020 [41]	1	Widespread	Leucocitoclastic vasculitis
Balestri et Al.,November 2020 [42]	1	Feet	Necrotic lesions
Rossi E et Al., November 2020 [43]	1	Widespread	Acute urticarial rash
Negrini S et Al., November 2020 [44]	1	Widespread	Bollous haemorragic vasculitis
Carugno A et Al., November 2020 [45]	1	Arms, trunk and lower limb	Erythemato-oedematous morbilliform rash
Proietti et Al., November 2020 [46]	1	Auricle	Perniosis
Annunziata et Al., November 2020 [47]	4	Trunk and limbs	Asymptomatic erythematous-pomphoid skin rash

Promenzio et Al., November 2020 [48]	4	Acral sites	Erythema pernio-like
Quaglino et Al., December 2020 [49]	1	Trunk and limbs	Macular rash with confluent erythema (before)<br>Livedo-like lesions (after)
Montinari et Al., January 2021 [51]	1	Abdomen and upper arms	Erythematous annular plaques with central clearing and peripheral scaling
Pezzarossa et Al., January 2021 [52]	12	Widespread	Acute Generalized Exanthematous Pustulosis (AGEP)
Patrì et Al., February 2021 [53]	2	Trunk,shoulders and neck<br>Trunk, upper limbs, neck and face	Purpuric eruptions (1 patient)<br>Sub-erythroderma (1 patient)

Abbreviations	Word
WHO	World Health Organization
Covid-19	Coronavirus Disease-19
PCR	Polymerase Chain Reaction
ACE2	Angiotensin conversion enzyme 2 receptor
ARDS	Acute Respiratory Distress Syndrome
RT-PCR	Reverse transcriptase polymerase chain reaction
H&E	Hematoxylin-Eosin
IHC	Immunohistochemistry
AGEP	Acute generalized exanthematous pustolosis
